# Pancreatic stellate cells in pancreatic cancer: as potential targets for future therapy

**DOI:** 10.3389/fonc.2023.1185093

**Published:** 2023-06-20

**Authors:** Zhengfeng Wang, Ru He, Shi Dong, Wence Zhou

**Affiliations:** ^1^ Department of General Surgery, The First Hospital of Lanzhou University, Lanzhou, China; ^2^ The Second School of Clinical Medicine, Lanzhou University Medical College, Lanzhou, China; ^3^ Department of General Surgery, Lanzhou University Second Hospital, Lanzhou, Gansu, China

**Keywords:** pancreatic cancer, pancreatic stellate cells, oxidative stress, molecular targets, drug theraphy

## Abstract

Pancreatic cancer is a strongly malignant gastrointestinal carcinoma characterized by late detection, high mortality rates, poor patient prognosis and lack of effective treatments. Consequently, there is an urgent need to identify novel therapeutic strategies for this disease. Pancreatic stellate cells, which constitute a significant component of the mesenchymal cellular layer within the pancreatic tumor microenvironment, play a pivotal role in modulating this environment through their interactions with pancreatic cancer cells. This paper reviews the mechanisms by which pancreatic stellate cells inhibit antitumor immune responses and promote cancer progression. We also discuss preclinical studies focusing on these cells, with the goal of providing some theoretical references for the development of new therapeutic approaches for pancreatic cancer.

## 1 Introduction

Pancreatic cancer (PC) is a highly lethal malignancy characterized by a proliferative response of bridging tissue leading to the emergence of a fibrotic stroma. This stroma not only provides a hypoxic micro-environment for tumor cells of tumor, but also creates a physical barrier that hampers the effective delivery of drugs are delivered to the tumor (1). PC is projected to become the second leading cause of cancer-related deaths in the US within the next 20 years to 30 years (2) and is expected to replace the third leading cause of cancer deaths across the European Union (3).

Given that most patients present with unresectable distant metastases at the time of diagnosis, the five-year survival rate is a mere ten percent. Even among the few patients who are eligible for resection, the prognosis remains poor, with a median survival of approximately 10-12 months post-treatment and only 20% of patients surviving for five years (1). Despite genetic and epigenetic research identifying key alterations driving the progression of PC, such as the mutations in Kras, p53, and SMAD4, none of these targets have resulted in effective therapies, making PC is still one of the most challenging cancers to treat (4). Therefore, the exploration of new therapeutic strategies is crucial to improve outcomes in cancer progression and metastasis.

Currently, tumor-mesenchymal interactions are now recognized as playing a vital role in the progression of PC, with the rich stroma being primarily generated by pancreatic stellate cells (PSCs) (5). Moreover, sustained activation of PSCs is a major contributor to fibrosis in pancreatic disease (6). There is increasing evidence that PSCs play a leading role in both normal and abnormal function. PSCs interact closely with various cells, such as endothelial cells and immune cells, to create an environment conducive to PC growth, thereby promoting the growth and metastasis. Therefore, developing therapeutic strategies targeting PSCs could potentially impede PC development (7).

## 2 Pancreatic stellate cells

### 2.1 Activation of pancreatic stellate cells

In the normal pancreas, PSCs are typically quiescent, residing around the alveoli as lipid storage cells, They are a crucial component of the tumor microenvironment, involved in maintaining pancreatic tissue structure and epithelial mesenchymal transition (EMT) (8, 9). Chronic inflammation, environmental pressure (such as oxidative stress), chronic smoking, increased secretion of IL-1, IL-6, TGF-β, as the upregulation of key pathways can lead to the activation of PSCs from their resting phase (10). Under hypoxic conditions, PSCs can be activated by oxidative stress induction, leading to pancreatic fibrosis (11).

Activated PSCs exhibit a myofibroblast-like phenotype, characterized by the expression of α-smooth muscle actin (α- SMA), production and proliferation of extracellular matrix (ECM) and, in particular, an increase in type I and IV collagen, laminin and fibronectin. The deposition of these elements results in significant interstitial fibrosis (12, 13). Activated PSCs can create a conducive micro-environment and promote cancer progression by altering four pathways in PC models (1): hyperfibrosis (2), promotion of tumor metastasis (3), induction of drug resistance, and (4) immune regulation (14).

Activated PSCs play a crucial role in PC by promoting tumor growth, invasive metastasis, immune escape, and inflammatory response (15). Additionally, PSCs stimulate angiogenesis, which is essential for tumor growth and metastasis, impairment of the antitumor immune mechanism, and indirect induction of immune cell dysfunction (16). One of the major factors contributing to PC treatment resistance is the fibroproliferative response induced by activated PSCs. This response increases pressure within the tumor and acts as a barrier to the tumor, restricting blood flow and the delivery of immune cells, therapeutic agents and oxygen to the tumor (17).

**Figure 1:**
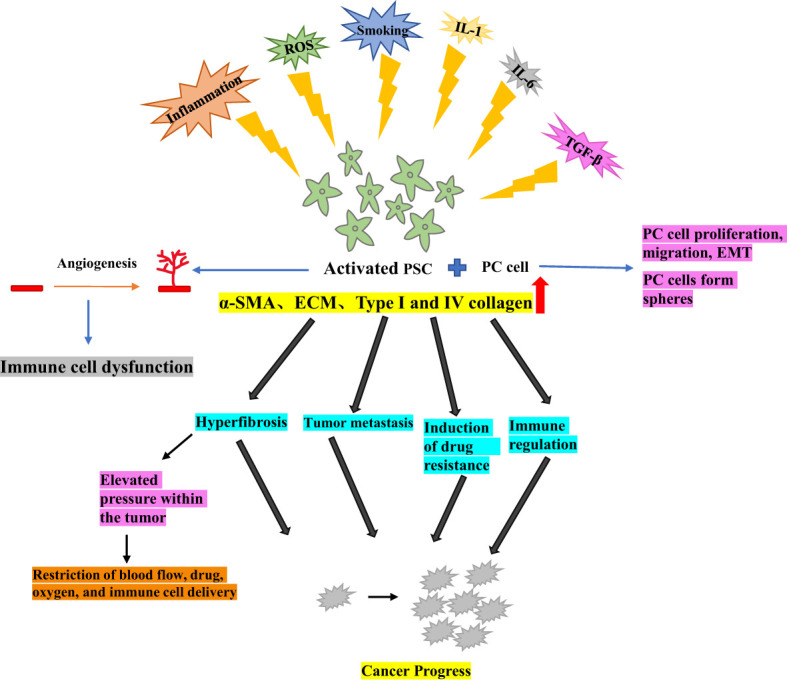
Activation of pancreatic stellate cells in pancreatic cancer.

Moreover, when PC cells were co-cultured with PSCs, PC cell migration, and EMT production were increased (18), and tumor cell spheroid formation sphericity was promoted, This suggests that PSCs could enhance the tumor stem-like phenotype of the PC cells (19). Therefore, further elucidation of the functional and clinical significance of PSCs could effectively guide the development of tumor therapy (**Figure 1**).

### 2.2 Pancreatic stellate cells and oxidative stress

Oxidative stress is a detrimental reaction resulting from an imbalance between oxidative and antioxidant mechanisms, triggered by free radicals, which instigates numerous diseases (20). Reactive oxygen species (ROS), for instance, contribute to organismal aging and disease development. PSCs play a pivotal role in pancreatic cancer fibrosis, a process intrinsically linked to oxidative stress. It has been discovered that drugs capable of regulating ROS not only selectively kill cancer cells but also modulate normal cell physiology and inflammatory diseases (21). PSCs can be activated under the influence of oxidative stress through the MAPK/AP-1 pathway, mediated by fibrillary regulatory protein (FMOD) (22). This activation of PSCs leads to islet fibrosis. One study found that high glucose levels escalated oxidative stress in PSCs, promoting cell activation and leading to an increase in pancreatic fibrosis (23). However, antioxidant treatment mitigated these changes. In a rat model, the antioxidant glutathione was found to inhibit PSC-activated pancreatic fibrosis by blocking ROS/TGFβ/SMAD signaling both *in vivo* and *in vitro* (24). Therefore, reducing oxidative stress production and PSC activation is key to halting the process of pancreatic fibrosis.

## 3 The role of pancreatic stellate cells in pancreatic cancer

### 3.1 Pancreatic stellate cells suppress antitumor immune response

Despite some progress in the development of immunotherapies for pancreatic cancer(PC), treatment has not yet shown significant results. Exploring the role of PSCs in the antitumor immune response may be a promising direction for the immunotherapy of PC. In pancreatic cancer, PSCs promote immunosuppressive elements within the tumor microenvironment and suppresses immune cell populations (25).

Firstly, in T cells, PSCs not only directly inhibit T cell infiltration, but cytokines secreted by tumor cells or PSCs also modulate T cell infiltration mediating immunosuppression. NFκB in PSCs increases CXCL12 expression thereby reducing cytotoxic T cell infiltration in the tumor and decreasing the killing effect on cancer cells, thus promoting tumor growth (26). In the presence of cancer-associated fibroblasts (CAF), the expressions of CD4 and CD8 T cell immune checkpoints are enhanced, leading to a decrease in immune function (27). In contrast, tumor cell-derived IL-1β regulates the activation and secretory phenotype of PSCs; besides, its oncogenic effects are mediated through the immunosuppression of CD8 T cell activity and infiltration (28). In a mouse model, it was discovered that the activated PSCs regulate chemokines, cytokines, and T cell adhesion molecules for migration, reduce the CD8 T cell migration to peritumoral tumor stromal compartments, thereby impeding their entry into cancer cells, and blocking antitumor immune responses (29).IL-6 and STAT3 secreted in the supernatant of PSCs boost the differentiation of peripheral blood mononuclear cells (PBMC) into myeloid-derived suppressor cells (MDSC) and inhibit autologous T cell proliferation. Conversely STAT3 inhibitors can abrogate this differentiation effect (30). In another study, PC cells and PSCs co-cultured with conditioned medium (CM) induced MDSC exhibited lymphocyte suppression. The lymphocyte suppression was weaker in PC cells of primary tumor origin than in PC of metastatic tumor origin.PSC-induced MDSC had a strong suppressive effect on Th2, whereas when co-cultured, it showed a strong suppression of Th1 (31). This suggests that the co-culture of these two cells enhances the suppression of the antitumor immune response.

In addition, there is a suppressive role of PSCs on the antitumor immune response in relation to other immune cells. Co-culture of PSCs and macrophages activate PSCs, promotes the formation of PC fibrosis, and increases the difficulty of cancer treatment (32). Subsequent studies have shown that mannose receptor (MRC1)-mediated collagen internalization and increased arginine levels lead to the regulation of inducible nitric oxide synthase and the generation of the reactive nitrogen species, enhancing collagen deposition, promoting a pro-fibrotic phenotype in PSCs, enhancing intra-tumor fibrosis, and increasing the difficulty of treatment (33).

Activated PSCs from the pancreatic tumor stroma has a negative impact on NK cells, which may be crucial for the suppression of the antitumor immune response of NK cells within the tumor microenvironment (34). Therefore, further exploration of the specific mechanisms of NK cell regulation by activated PSCs could provide new insights into PC therapy. It was also found that peptidylarginine deiminase 4 (PADI4) deficiency transplanted into Kras-driven pancreatic adenocarcinoma (Pdx1-Cre : Kras) mice -/- G12D/+, DNA released from Neutrophil extracellular trap activated PSCs, formed a dense fibrous stroma, and promoted tumor growth. Furthermore, the deletion of late glycosylation end-product receptors in the PSCs eliminates the role of DNA in promoting stellate cell proliferation and reducing tumor growth (35). This finding supports the investigation of Neutrophil extracellular trap, extracellular DNA and PADI4 as possible therapeutic strategies for patients suffering from PC.

Regarding immunomodulation in PC, PSCs appear to be strong immunosuppressive manipulators through multiple pathways. By targeting PSCs, researchers might explore an emerging way of improving the antitumor immune response in PC.

### 3.2 Specific mechanisms by which pancreatic stellate cells promote the progression of pancreatic cancer

#### 3.2.1 Anabolic

Cells of PC actively proliferating in the tumor microenvironment have increased glutamine uptake and dependence (36). Glutamine anabolic pathways are higher in PSCs compared to PC cells, and PSCs promote β-catenin/Wnt/TCF7-mediated glutamine synthetase (GS) with the aim of boosting glutamine synthesis to accelerate PC cell proliferation and tumor growth *in vivo* (37). This suggests that stroma-associated PSCs are crucial for the metabolism of PC by secreting nonessential amino acids. It has also been found that under nutrient-limited conditions, PSCs rely on autophagy-derived protein-derived alanine to promote lipid biosynthesis and nonessential amino acid production, thereby promoting cancer cell growth within the tumor microenvironment (38). Tumor-derived IL-17B interacting with extracellular bursas causes the expression of IL-17RB in PSCs, reduces mitochondrial fission, increases oxidative phosphorylation, supports pancreatic cancer growth, and accelerates tumor growth in xenograft mouse models. Activated tumor cells in the feedback loop decreased glycolysis by increasing oxidative phosphorylation *via* IL-6. The results suggest that under optimal nutritional conditions, the tumor-to-stroma feedback circulation increases the tumor metabolism and accelerates tumor growth (39).

#### 3.2.2 Co-culture system

Co-culture of PSCs with PC cells has been found to promote tumor progression *via* multiple pathways. Co-culture of tumor-associated PSCs with PC cell lines showed reduced E-calmodulin levels, adjustment of Vimentin, and decreased levels of the tight junction protein ZO-1, suggesting that PSCs induce EMT changes in PC cells that promote invasion (40). It was also found that mediating NGF/TrkA activation of the GSK/AKT/PI3K signaling cascade response within the co-culture system promotes the invasive and proliferative capacity of pancreatic cancer cells (41). Within the tumor microenvironment, TrkA/NGF was shown to be a potential and effective treatment target for patients with PC. PSCs reconstitute the actin cytoskeleton through Endo180-myosin light chain 2 (MLC2) signaling. Invasion of the extracellular matrix (ECM) attenuates the aggressive capability of co-cultured PC cells (42). In other co-culture systems it was identified that cancer cell-derived PAI-1 mediated by KRAS can activate PSCs *via* IL-8 and aggravate the malignant action of cancer cells. Conversely inhibition of IL-8 signaling reduces pancreatic tumor growth and fibrosis *in vivo* (43). CaPSCs isolated from PC patients and PSCs isolated from benign patients and fostered with the cancer cell line PANC-1 revealed that Chr7:154954255-154998784+ might boost the development of PC by means of the miR-4459/KIAA0513 axis within CaPSCs and become a pivotal target for the treatment of PC patients in the future (44). PSC co-culture with PC cells induced increased GPR68 expression, improved IL-6 expression *via* the cAMP/PKA/cAMP response element binding protein signaling pathway, increased fibrosis markers, and IL-68 production to promote PC cell proliferation. Among others, GPR68 is a mediator of low pH promoting tumor microenvironment regulation, especially in PC-CAF interactions, and may become a new treatment target for the pancreatic carcinoma and other types of carcinomas (45).

#### 3.2.3 Autophagy and senescence

Autophagy is PSC activation linked to the activation of PSCs and the progression of PC. Clinical samples have shown higher levels of autophagy markers in PSCs from pancreatic tumor samples. PSC exposed to autophagy inhibitors formed smaller tumors with fewer metastases of the liver and less peritoneal spread in nude mice. Autophagic PSCs produce ECM and IL-6, which are linked to a shorter survival time and disease recurrence among PC patients (46). It has also been found that senescent PSCs boost the migration and proliferation of MIAPaCa-2 cell lines and pancreatic carcinoma AsPC-1 *via* the CXCL1/CXCR2 axis, where CXCL1, CXCL2 and CXCL3 are senescence-related secretory phenotypic elements secreted from the senescence-induced PSCs, and CXCL1/CXCR2 axis antagonists attenuate their migration and proliferation of pancreatic cancer cell stimulatory effects. These findings support the idea that the senescent PSCs in the PC tumor microenvironment are pro-cancerous and could contribute to the development of new treatment agents for PC (47). Dissection of PC patient samples revealed that Sequestosome-1 (sqstm1) expression was reduced in the activated PSCs, which promoted the pancreatic tumor cell growth, aggression and macrophage phenotype conversion through NRF2/ROS regulation of the senescent and inflammatory phenotype of the pancreatic stellate cells. Meanwhile enhanced autophagy-induced sqstm1 degradation was not connected to the conversion of PSCs senescent phenotype (48).

#### 3.2.4 Exosomes

Exosomes interact with stromal components and PC cells, such as PSCs, in the tumor microenvironment to regulate the progression of PC. It has been shown that the PSC-derived exosome miR-5703 downregulates CKLF, including MARVEL transmembrane domain containing 4 (CMTM4) in PC cells with the aim of promoting cancer cell proliferation through PAK4 activation of the Akt/PI3K pathway. Furthermore, it also suggests that serum exosome miR-5703 may serve as a diagnostic biomarker for PC diagnosis (49). It has also been found that exosomes miR-616-3p and miR-4465, produced by PSCs in a hypoxic environment, are upregulated and inhibit the AKT/PTEN pathway with the aim of promoting cancer cell metastasis and progression (50). PSCs, which are typically activated in PC, release exosome miR-21, promoting cancer cell migration and EMT, as well as enhancing the activity of the ERK/Ras signaling pathway (51). These findings suggest that PSC-derived exosomes may have a predictive value for poor prognosis in patients with PC and could be a new target for cancer therapy.

#### 3.2.5 Extracellular matrix

One of the main challenges in treating PC is the complex interaction between stromal components, cell-cell communication with each other, and the secretion of factors that promote cancer growth. Studies have investigated the paracrine secretion between PSCs and PC cells and identified leukemia inhibitory factor (LIF) as a key paracrine factor that acts on PC cells. LIF regulates cancer cell differentiation and EMT, and plays a crucial role in tumor chemoresistance and progression (52). PSCs in the stroma secrete important ligands such as Wnt and tenascin C (TnC), which act in a paracrine manner on PC cells to activate oncogenic β- catenin and YAP/TAZ signaling pathways, promoting tumorigenic behavior. However, the N-myc downstream regulatory gene-1 (NDRG1) targets the Wnt/TnC-mediated interaction between stellate cells and PC cells to inhibit cancer progression (53). It has been reported that ATRA, the active vitamin A metabolite, restores the quiescence of PSCs through retinoic acid receptor β (RAR-β)-dependent actin (MLC-2) contractility. ATRA reduces the excessive traction force generated by PSCs and their ability to remodel the extracellular, inhibits ectocytic matrix modeling again thereby locally suppressing carcer cell aggression in a three-dimensional organotypic model. This suggests that PSC reprogramming with retinoic acid may be a promising approach for treating pancreatic carcinoma (54).

#### 3.2.6 Target genes

Elevated expression of NF-E2-related factor 2 (Nrf2) has been observed in PC. Conditioned media (CM) derived from Nrf2-deficient PSCs showed reduced growth stimulation in PC cells. In mouse models, co-injection of Nrf2-deficient PSCs with KPC mouse-derived pancreatic cancer cells resulted in the formation of smaller subcutaneous tumors compared to wild-type PSC co-injections (55). Both human and murine PSCs express the P2X7 receptor (P2X7R), which influences P2X7R activation and leads to the release of collagen and IL-6. P2X7R activation also stimulates JAK/STAT3 signaling, contributing to oncogenic effects (56). Kindlin-2, expressed in both activated PSCs and PC cell, is found to be expressed more in PSCs and correlates with a shorter relapse-free recurrence survival time. Knockdown of Kindlin-2 in PSCs reduces migration and proliferation were reduced in PSC and inhibited in PSCs and inhibits PC cell growth. Tumor support is also eliminated in nude mice (57). Bcl2-associated athanogene3 (BAG3) expression is upregulated in activated PSCs. BAG3 secretes TGF-β2, IL-6, and IGFBP2, which not only to maintain activation of PSCs but also to promote migration and invasion of PC cells (58). ITGA11 expression was found to be upregulated in primary of ITGA11 in PSCs activated by PANC-1 conditioned media or TGF-β. Knockdown of ITGA11 in PSCs resulted in reduced tumor cell invasion and migration, indicating its important role in PSC differentiation to cancer-associated fibroblasts (CAF) and paracrine effects (59). Yes-associated protein 1 (YAP1) is highly expressed in the core of PC-derived activated PSCs. Knockdown of YAP1 expression or pharmacological inhibition of YAP1 leads to PSC inactivation and inhibition of PC cell proliferation. Targeting YAP1 provides new insights into reprogramming the tumor microenvironment (60). High CD51 expression in the PC stroma has been associated with lymph node metastasis of the lymph node, active pathological margins, and reduced patient survival. Knockdown of CD51 in PSCs impedes the tumor stroma and reduces cancer cell proliferation, suggesting CD51 as a potential therapeutic target for the pancreatic carcinoma (61). In conclusion, these target genes and molecules involved in thecrosstalk between cancer cells and PSCs may offer potential therapeutic strategies to inhibit tumor progression. Inhibiting the interactions and signaling pathways between PSCs and cancer cells could be a promising approach for the treatment of pancreatic carcinoma.

#### 3.2.7 Other

**Figure 2:**
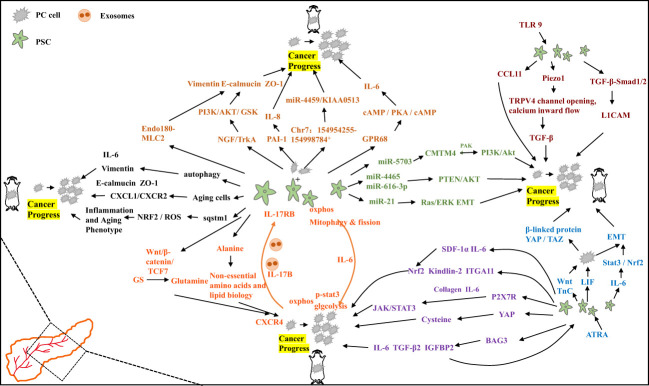
Specific mechanisms by which pancreatic stellate cells promote the progression of pancreatic cancer.

Activation of Toll-like receptor (TLR) signaling has been shown to have differential effects on tumorigenesis development. In PC, TLR9 activation induces PSC fibrosis and in PSCs promotes the secretion of the chemokine CCL11, which maintain a pro-inflammatory tumor microenvironment and promotes tumorigenesis (62). Fibrosis in PC is characterized by the excessive production of ECM by activated PSCs. Elevated intraductal pressure also contributes to PSC-mediated pancreatic fibrosis. Studies have revealed that PSC activation involves the mechanically activated ion channel Piezo1, which initiates the fibrotic response and triggers the opening of the TRPV4 channel, leading to calcium influx. This process is accompanied by increased expression of TGF-β, which contributes to chronic pancreatitis and fibrosis (63). Overexpression of H,K-ATPase (encoded by ATP12A and ATP4A) has been observed in PC and PSC cells in both human and murine models. Proton pump inhibitors have been found to reduce collagen secretion from PSCs, leading to decreased fibrosis and tumor growth. This suggests that H,K-ATPase plays a role in pancreatic cancer progression and provides a potential therapeutic target (64). PSC secreted TGF-β1 passively regulates the expression of L1 cell adhesion molecule (L1CAM) through *via* TGF-β-Smad1/2 signaling. This promotes PC cell stemness, increases PC cell invasiveness, and provides new insights into tumor suppression (65). Furthermore, PSCs secrete stromal cell-derived factor-1α (SDF-1α) and IL-6, which induce pancreatic cancer cell proliferation through Nrf2-activated metabolic reprogramming and ROS detoxification. IL-6 also promotes epithelial-mesenchymal transition (EMT) in pancreatic ductal adenocarcinoma (PDAC) cells *via* the Stat3/Nrf2 pathway (66, 67). The mechanism diagram illustrating these interactions is shown in **Figure 2**.

## 4 Drugs targeting pancreatic stellate cells for the treatment of pancreatic cancer

Due to of the role of PSCs in pancreatic cancer, drugs that affect its activation are potential candidates for the treatment of pancreatic cancer, as shown in **Table 1**.

**Table 1:** Drugs targeting pancreatic stellate cells for the treatment of pancreatic cancer.

Drugs	Action on PSC activity	Mechanism	References
Glutathione	Inhibition	ROS/TGFβ/SMAD	(23)
GEM	/	Reduction of the effect of GEM and other nucleoside analogues on cancer cells	(63)
	Inhibition (novel peptidomimetic AV3)	Reduces fibroplasia and enhances tumor perfusion	(64)
	/	ERK phosphorylation, enhanced glycolysis	(65)
	/	activation of ERK1/2	(66)
	/	Polyethylene glycolated nanocoupling ACG44P1000 delivery vector for enhanced cytotoxicity	(67)
	Inhibition	ATO nanoparticles target PI3K/AKT/AP4/galactose lectin-1	(68)
	Inhibition (combined with PTT)	TGF-β and collagen fibril expression	(69)
Paclitaxel	Pancreatic tumor spheroids co-cultured with PSC	Combination of paclitaxel and gemcitabine to study drug resistance	(70)
Tamoxifen	Inhibition	Reduction of macrophage recruitment and polarization GPER reprogramming of PSC	(71)
Nanoparticles	Inhibition	Combined with gemcitabine significantly inhibited tumor progression	(72)
Combination of antifibrotic drugs and iron death inducers	/	Refractory PC iron death	(73)
EGFR inhibitor and Met inhibitor combination	/	HIF-1α-HGF-Met-PI3K-AKT signaling axis	(74)
Combination of NAC and pioglitazone	Inhibition	Reduction of oxidative stress levels Enhanced chemosensitivity of PC cells	(75)
RSV	Inhibition	ROS/miR-21 activation and glycolysis	(76)

### 4.1 Gemcitabine

Gemcitabine (GEM) is an effective treatment for PC, but resistance the drug can develop. The understanding of the relationship between drug resistance and the stroma is crucial. PSC-derived conditioned media (CM) has been shown to inhibit the processing of GEM in cancer cells by regulating the secretion of deoxycytidine through nucleoside transport proteins, This reduces the impact of GEM and other nucleoside analogues on cancer cel, making them resistant to GEM toxicity (68). Integrin α5 (ITGA5) is overexpressed in PC patient samples and is negatively correlated with overall survival. ITGA5 induces PSC activation through the TGF-β/Smad2/FAK pathway. A novel mimetic peptide of ITGA5, AV3, has been found to reduce fibroplasia, improve tumor perfusion, inhibit PSC activation, and enhance the effectiveness of GEM in xenograft tumor models within a 3D spheroid model (69). Factors secreted by PSCs mediated ERK phosphorylation, enhance PKM2 phosphorylation, and increase the expressions of MCT4 and LDHA, leading to intensified glycolysis and decreased sensitivity to GEM. Glycolysis may contribute to chemotherapy resistance in pancreatic cancer (70). PSCs have been found to induce varying levels of resistance to gemcitabine cytotoxicity in cancer cells in both direct and indirect co- culture systems, PSC secreted fibronectin (FN) in ECM plays a key role in GEM resistance through the activation of ERK1/2. Blocking FN in combination with GEM chemotherapy may reduce resistance and improve clinical outcomes (71). Nanotherapeutics using low molecular weight polyethylene glycolized nanocouple ACG44P1000, targeted to EGFR, showed enhanced cytotoxicity to pancreatic cancer cells and PSCs, resulting in *in vitro* more pronounced tumor regression and less toxicity to healthy tissue (72). Targeting the AP4/AKT/PI3K galectin-1 pathway with arsenic trioxide (ATO) nanoparticles targeting AP4/AKT/PI3K galactose lectin-1 pathway inhibited its activation, reduced stromal secretion, attenuated the tumor-promoting capability of PSCs, and enhanced the sensitivity of cancer cells to GEM. This offers a new perspective on PC treatment (73). Combining photothermal therapy (PTT) to locally consume the stroma, intensify GEM permeability, and synthesize C-G NPs has shown promise in inhibiting PSC activation, collagen fibers, and TGF-β expression. This holds great potential for PC treatment (74). These findings not only provide insights into GEM resistance in PC, but also offer some promising therapeutic strategies to inhibit PC growth. They have the potential for future clinical translation.

### 4.2 Paclitaxel

PC cells, specifically PANC-1 cells, formed tumor spheroids within three days. The addition of PSCs to the culture increased the quantity of spheroids. Treatment with gemcitabine alone did not significantly affect survival. However, when paclitaxel was combined with gemcitabine, there was a significant inhibition of tumor spheroid growth, induction of EMT, increased drug sensitivity, and cytotoxicity in PSCs. This suggests that co-culturing pancreatic tumor spheroids with PSCs can serve as an effective model for studying drug resistance (75). In a preclinical model, targeting PSCs with ATRA led to reprogramming of the stroma in the pancreas, resulting in the inhibition of PC growth. The use of ATRA as a stromal targeting agent is currently being evaluated in a phase II randomized controlled trial for locally advanced PC (76).

### 4.3 Tamoxifen

Tamoxifen has been shown to reduce the contractility of myofibroblasts, which are activated PSCs, and inhibit their ability to deform the underlying matrix, thus reducing fibrosis. This suggests that tamoxifen may play a role in regulating PSC myofibroblast activation (77). In addition to its effects on myofibroblasts, tamoxifen has been found to reduce macrophage recruitment and polarization. It inhibits macrophage spreading, cell-matrix attachment, and invasion. Tamoxifen also inactivates PSCs, increases matrix stiffness to promote the inactivation of YAP, and acts through the G protein-coupled estrogen receptor (GPER) to inhibit fibrosis in a mouse model of PC. Furthermore, tamoxifen regulates the immune response and impedes ECM remodeling and cancer cell aggression by reprogramming the PSCs through GPER and inhibiting myofibroblast differentiation, and reducing their matrix remodeling capacity (78).

### 4.4 Nanoparticles

In the context of PC, various drug delivery systems using polymers and nanocarriers have been developed. One such system involves the use of poly(lactic-co-glycolic acid) (PLGA) nanoparticles as carriers loaded with chloroquine (Nano-CQ) or indocyanine green (Nano-ICG). These nanoparticles have shown promise in delivering drugs to pancreatic tumors. When administered, Nano-ICG accumulates in pancreatic tumors and areas of peritoneal metastases, while sparing normal tissues. This targeted delivery system allows for precise drug delivery to the tumor site. In addition, lower doses of chloroquine (CQ) loaded in the nanoparticles have been found to reduce the number of activated PSCs and inhibit tumor progression when combined with gemcitabine, a commonly used chemotherapy drug. This suggests that PLGA nanosystems can effectively deliver drugs into pancreatic tumors and may serve as a promising pretreatment method for pancreatic cancer (79).

### 4.5 Other

In the context of pancreatic cancer (PC), activated PSCs play a significant role in promoting tumor progression and resistance to therapy. Several studies have investigated potential therapeutic approaches targeting PSCs to improve treatment outcomes. One strategy involves targeting the HGF-Met signaling axis, which is activated by growth factor HGF secreted by activated PSCs. This signaling pathway contributes to PC resistance to iron-induced cell death. Combining antifibrotic drugs with iron death inducers has shown potential in promoting iron-induced cell death in refractory PC, providing a potential clinical therapy approach (80). Hypoxia has also been identified as a factor that reduces the sensitivity of PC cells to EGFR inhibitors through the HIF-1α-HGF-Met-PI3K-AKT PSC signaling axis. Inhibition of both Met and EGFR signaling has demonstrated inhibitory effects on tumor growth in preclinical models, suggesting a promising therapeutic strategy for PC (81). The expression of p-ERK1/2 has been found to be increased in cancer-associated PSCs and is associated with PC progression. Inhibition of p-ERK1/2 expression has shown potential in reducing PSC viability, inhibiting cancer-stromal interactions, and suppressing metastasis, highlighting its role in PC progression (82). N-acetylcysteine (NAC), a compound with antioxidant properties, has demonstrated inhibitory effects on PSC viability, migratory capacity, and invasiveness. Additionally, it reduces oxidative stress levels, and attenuates cancer-stromal interactions. Combination therapy using NAC and pioglitazone, a drug used for diabetes treatment, has shown benefits in maintaining resting PSCs, enhancing the chemosensitivity of PC cells, and inhibiting tumor growth *in vitro* (83). Furthermore, natural phytochemicals such as resveratrol (RSV) have shown potential as therapeutic agents for PC. Resveratrol (RSV) is one polyphenol with powerful anticancer and antioxidant outcomes. In PC RSV inhibits PC cellular migration and aggression by suppressing PSC-mediated ROS/miR-21 glycolysis and activation. Exploring the role of natural compounds in cancer may be an emerging tactic for prevention and therapy, including PC, represents an emerging approach (84).

## 5 Discussion

PSCs play a crucial role in tumor-stroma interactions and contribute to tumor progression in pancreatic cancer (PC). PSCs promote cancer growth of cancers, metastasis, and resistance to chemo- or checkpoint inhibitory treatment through various mechanisms, including immunosuppression, angiogenesis, extracellular matrix remodeling, chemokines, secretion of tumor-promoting cytokines, and growth factors, and chemokines. Understanding the role of PSCs in PC and their impact on the antitumor immune response is essential for improving PC treatment. Targeting PSC activation has become a focus of research to develop novel therapeutic strategies. By inhibiting or reversing PSC activation, it is possible to disrupt the tumor-promoting interactions between PSCs and PC cells, thereby slowing down cancer progression. Additionally, studies have shown that PSCs also play a role in endocrine cell function, diabetes, and islet fibrosis, further highlighting the significance of understanding PSC biology. Advancements in technology have provided valuable insights into the pathways involved in PSC activation, offering potential therapeutic targets to interfere with PSC activation and disrupt PSC-PC cellular interactions. Immortalization of PSCs has facilitated their study and enabled a deeper understanding of the molecular mechanisms underlying pancreatic fibrosis. These advancements have paved the way for the development of new strategies for targeting PC therapy. However, there is still much to be learned about the complex mechanisms of PSC activation and the molecular processes driving pancreatic fibrosis. Further research is needed to unravel these complexities and develop innovative approaches for targeting PC therapy. Continued efforts in studying PSCs and their interactions with PC cells hold the promise of significant breakthroughs in the treatment of pancreatic diseases.

## Author contributions

Z.W.: Writing-Original draft preparation, Supervision, Project administration, R.H. and S.D.; Writing - Review & Editing, Supervision. W.Z.; Writing-Original draft preparation, Supervision, Project administration, Funding acquisition. All authors contributed to the article and approved the submitted version.
